# Transapical aortic valve implantation in patients with pre-existing mitral valve prostheses: a case report

**DOI:** 10.1186/s13019-016-0521-0

**Published:** 2016-08-08

**Authors:** Kristina Wachter, Samir Ahad, Christian J. Rustenbach, Ulrich F. W. Franke, Hardy Baumbach

**Affiliations:** Department of Cardiovascular Surgery, Robert-Bosch-Hospital, Auerbachstr. 110, D-70376 Stuttgart, Germany

**Keywords:** Heart valve prosthesis, Transcatheter aortic valve replacement, Mitral valve, Minimally invasive surgical procedures

## Abstract

**Background:**

Transcatheter aortic valve implantation (TAVI) has proven to be a valid option for patients with severe aortic stenosis who are at high perioperative risk, particularly in patients with previous cardiac surgery. Several patients with previous mitral valve surgery were reported to have been successfully treated with TAVI.

**Case presentation:**

Two patients, one with mechanical and one with biological mitral valve prosthesis, presented with symptomatic severe aortic stenosis. After discussion among our multidisciplinary heart team transapical approach and a JenaValve™ prosthesis was used for TAVI. Main reasons were to decrease the perioperative risk, avoid a re-opening of the chest via median sternotomy, and discuss the possible superiority of the JenaValve™ device due to its design. The patients were successfully treated and discharged on the 11th and 14th post-operative day, respectively. Echocardiographic follow up before discharge and up to 2.8 years post-operatively showed excellent results.

**Conclusions:**

In conclusion, TAVI in patients with preexisting mitral prostheses-mechanical or biological-is feasible, safe, and effective and offers a valid alternative to conventional aortic valve replacement in this particular re-operation scenario. The JenaValve™ device does not interact with the mitral prosthesis and offers therefore due to its unique design a potential advantage.

## Background

Considerable proportion of patients who require mitral valve replacement (MVR) presents with a coexisting pathology of the aortic valve [1, 2] with a possible necessity of surgery of the aortic valve in the following years. On the other hand, the perioperative risk of morbidity and mortality is elevated in patients undergoing conventional aortic valve replacement with previous median sternotomy [3].

Transcatheter aortic valve implantation (TAVI) is nowadays an approved treatment for aortic stenosis (AS) in patients who are at high surgical risk [4] and it can further reduce perioperative risk especially in patients who had undergone previous cardiac surgery as there is potentially less surgical trauma [5]. Nevertheless, previous coronary artery bypass grafting (CABG), aortic valve replacement (AVR) or mitral valve replacement pose unknown risks when TAVI is performed.

We report two cases of severe AS treated by transapical TAVI in patients who underwent previously MVR with a mechanical and biological valve, respectively.

## Case presentation

The first case is a 76 year old patient presented to our hospital with progressive dyspnea, currently NYHA class III (New York Heart Association) and recurrent cardiac decompensation with a right pleural effusion and consecutive dystelectasis. Diagnostics, including transthoracic (TTE) and transesophageal echocardiography (TEE), revealed a severe aortic valve stenosis (Δp_mean_ = 83 mmHg, effective orifice area (EOA) = 0.4 cm^2^) [6]. The selective coronary angiography identified a coronary sclerosis without significant stenosis. In consequence to his severe mitral valve vitium the patient received a 27 mm bileaflet mechanical mitral valve prosthesis (Carbomedics, Sorin Group, Milano, Italy) 17 years prior to the current symptomatic episode. Echocardiographic survey showed no signs of malfunction of the mitral valve prosthesis. Due to the medical history including chronic atrial fibrillation and mechanical mitral valve prosthesis the patient received anticoagulant therapy (vitamin K antagonists). Additional comorbidities are listed in Table 1. The patient was suffering from pre-renal, recently compensated chronic kidney insufficiency and had a history of duodenal ulcers, ischemic colitis, and bladder carcinoma.

**Table 1:** Preoperative demographics

	Patient 1	Patient 2
Age [y]	76	74
Sex	Male	Male
EOA [cm^2^]	0.4	0.7
EOAI [cm^2^/m^2^]	0.21	0.32
Pre-op ΔP_mean_ [mmHg]	83	35
LVEF [%]	61	65
NYHA-class	III	III
NTproBNP [pg/ml]	1086	2461
CCS	I	II
CABG	No	Yes
MVR	Mechanical Valve (Carbomedics, 27 mm)	Biological Valve (Perimount Plus, 27 mm)
Chronic atrial fibrillation	Yes	Yes
COPD [GOLD]	IV	III
PHT	Moderate	Severe
PAD [Fontaine]	II	II
EuroSCORE I [%]	32.83	55.56
EuroSCORE II [%]	11.61	11.08
STS-score, PROM [%]	5.52	6.72

(*CABG* coronary artery bypass grafting, *CCS* Canadian Cardiovascular Society, *COPD* chronic obstructive pulmonary disease, *EOA* effective orifice area, *EOAI* effective orifice area index, *EuroSCORE* European System for Cardiac Operative Risk Evaluation, *LVEF* left ventricular ejection fraction, *MVR* Mitral valve replacement, *NYHA* New York Heart Association, *PAD* peripheral arterial disease-fontaine classification, *PHT* pulmonary hypertension, *Pre-Op Δp*_*mean*_ preoperative mean transaortic pressure gradient, STS-score, *PROM* Society of Thoracic Surgeons-score, predictive risk of mortality, *y* years)

The second case is a 74 year old patient complaining about progressive dyspnea (NYHA III) and episodes of stable load-dependent angina pectoris. Diagnostics also identified a severe aortic valve stenosis (TTE: Δp_mean_ = 41 mmHg; cardiac catheter: Δp_mean_ = 35 mmHg, EOA = 0.7 cm^2^ (according to Gorlin formula)) and coronary angiography showed still open bypasses after coronary artery bypass grafting in 2011. Due to chronic atrial fibrillation the patient received therapy with vitamin K antagonists as well. In addition to the preoperative demographics (Table 1), the patient received a pacemaker 4 years ago, because of bradyarrhythmia and was suffering from chronic lymphatic leukemia, recently in remission. In contrast to the first case, this patient received a 27 mm biological mitral valve prosthesis (Perimount Plus, Carpentier-Edwards, Irvine, USA) in 2011 with still excellent function.

Preoperative risk evaluation of perioperative mortality using the EuroSCORE (European System for Cardiac Operative Risk Evaluation) and the STS-Score (Society of Thoracic Surgeons) showed a high perioperative risk for both patients (Table 1). Furthermore, the patients strictly denied surgical aortic valve replacement via median sternotomy.

**Fig. 1:**
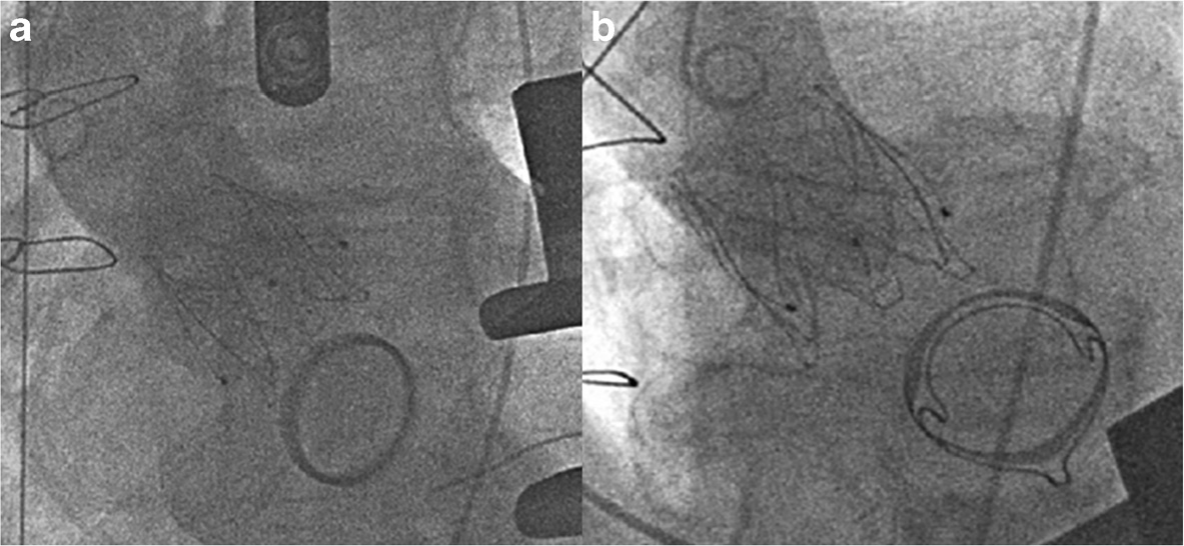
TAVI in a patient with **a** mechanical mitral valve prosthesis and **b** biological mitral valve prosthesis

The cases were discussed by our multidisciplinary team of interventional cardiologists and cardiac surgeons as recommended [7] and after considering preoperative diagnostics and all available treatment options, a transapical approach for transcatheter aortic valve implantation was favored. The annulus size of the aortic valve as well as the optimal angulation was determined preoperatively with gated CTA (Computed Tomography Angiography). The procedure was performed in general anesthesia in a fully equipped hybrid operating room as described previously [8]. An anterolateral approach via the 5th intercostal space was used for exposure of the apex. Rapid pacing was applied for balloon aortic valvuloplasty with a 22 mm balloon (NuCLEUS™, pfm medical AG, Cologne, Germany). A self-expandable prosthesis (27 mm JenaValve™, JenaValve Technology GmbH, Munich, Germany) was implanted in typical manner after positioning with optimal angulation without rapid pacing or hemodynamic instability. Neither transesophageal echocardiography nor aortography identified a para- or transvalvular regurgitation in both cases (Fig. 1a, b). The patients were either extubated in the operating room or shortly after being transferred to the intensive care unit for further monitoring. Patient one was discharged on the 11th postoperative day (POD), patient two on POD 14. Follow up examinations (before discharge, in the rehabilitation facility and 2.8 or 1.3 years postoperatively, respectively) were performed and both patients presented with dyspnea according to NYHA II without echocardiographic evidence of paravalvular leakage at each point in time (Table 2). In the last examination, mean transaortic pressure gradient was 17 and 18 mmHg, respectively.

**Table 2:** Intra- and postoperative data

	Patient 1	Patient 2
Skin-to-skin time [min]	63	52
Ventilation time [h]	10	0
ICU-stay [d]	3	1
Total hospital stay [d]	11	14
Aortic regurgitation discharge	None	None
Paravalvular leakage discharge	None	None
ΔP_mean_ [mmHg] discharge	10	14
NYHA-class discharge	II	II
Paravalvular leakage follow up	None	None
ΔP_mean_ [mmHg] follow up	17	18
Mitral prosthesis dysfunction follow up	None	None

*d* days, *h* hours, *ICU* intensive care unit, *NYHA* New York Heart Association, *ΔP*_*mean*_ mean transaortic pressure gradient

## Conclusions

After a few reports about implanting JenaValve™ in patients with mechanical mitral valve prosthesis [9, 10], we report here a case of transcatheter aortic valve implantation using the self-expandable JenaValve™ in a patient with history of biological mitral valve prosthesis.

Patients with symptomatic aortic valve stenosis that qualify for transcatheter implantation with pre-existing both, biological or mechanical mitral valve prostheses, are still a rare but increasing entity [1]. On the other hand, the perioperative risk of morbidity and mortality is elevated in patients undergoing conventional aortic valve replacement with previous median sternotomy [3]. Since TAVI-procedures were performed patients with pre-existing heart valve prostheses can be offered a new valid therapy option.

The first case of AS treated by TAVI in a patient with previous MVR was reported by Rodes-Gabau in 2008 [11]. Since then further publications reported the use of transapical [9–15], transfemoral [15–20] and even direct aortic [19] approaches to replace a severely stenotic aortic valve following MVR with different types of mechanical and biological mitral valve prostheses [9–21] or mitral valve reconstructions [19]. Possible complications, the risk of embolization or interference due to the mitral prosthesis’ struts, may complicate those procedures [13, 17]. Therefore, such patients were excluded from the Partner trial [22] and Medtronic CoreValve U.S. Pivotal Trial [23].

In contrast to the right ventricle with its dedicated outflow tract the left ventricle has a common aortic-mitral orifice with a close anatomical and physiological relationship between the aortic and mitral valve. The presence of a prosthetic mitral valve reduces the aortic-mitral distance and can therefore complicate an aortic valve implantation [24]. Additionally, the presence of a rigid mechanical structure instead of fibrotic tissue contributes to aggravate the situation [12]. These two mechanisms are the main causes for insufficient opening of the transcatheter valves, dislocation [13] or embolization [11]. Thus, despite good positioning of the CoreValve® prosthesis (Medtronic Inc., Minneapolis, USA), it can interfere with the opening of the mitral prosthesis’ leaflets and cause a life-threatening situation [17]. Yet an excessively high implantation can lead to aortic regurgitation or even worse occlusion of the coronary arteries [25]. Even dislocating of the Edwards Sapien® (Edwards Lifesciences, Irvine, CA) aortic valve prosthesis into the left ventricle 2 weeks after implantation has been reported [13].

In pre-existing biological mitral valve prosthesis, which have a different configuration compared to mechanical mitral valve prosthesis with more prominent commissural struts reaching into the left ventricular outflow tract (LVOT), TAVI procedure is even more challenging. Balloon displacement toward the aorta during inflation and valve malposition or embolization has been reported when implanting balloon expandable prostheses [12].

A minimum distance between the mitral valve prosthesis and the aortic annulus is recommended in both, self-expanding and balloon expandable TAVI to avoid a potential mitral valve dysfunction and to allow the correct expansion of the aortic valve prosthesis [20, 26]. Therefore, preprocedural screening of the patients and particularly the evaluation of mitro-aortic distance should be done precisely by multislice computed tomography [20]. Preoperative and intraprocedural transesophageal echocardiography, as well as fluoroscopy is also essential, to ensure a careful assessment of the patients’ anatomy and to monitor a precise device deployment.

According to our experience, the JenaValve™ is more securing in this setting. Because the locators of the JenaValve™ are positioned into the nadir of the aortic valve sinus the lower margin does not reach more than 2 mm into the LVOT below the aortic annulus and thus offering a reasonable safe distance that is needed to prevent interference with the mitral valve prosthesis during deployment [9]. The possibility of recapturing and repositioning of the device during deployment is also one major advantage to ensure optimal positioning of the prosthesis. Furthermore, a shorter valve length will prevent asymmetrical deployment thus decreasing the risk of paravalvular leakage [9].

For choosing a transapical versus a transfemoral approach, recommendations should be followed [27], yet a transapical approach was suggested to be more advantageous [12] due to more efficient prosthesis maneuvers.

Our report and the previous experience with JenaValve™ [9, 10] suggest that the JenaValve™ prosthesis may offer a potential advantage over other prostheses, due to its design, when implanted in patients with previous mitral valve replacement. However, larger series are needed to proof the anticipated superiority of the JenaValve™ device over other prosthesis.

## Abbreviations

AS, aortic stenosis; AVR, aortic valve replacement; CABG, coronary artery bypass grafting; CTA, computed tomography angiography; EOA, effective orifice area; EuroSCORE, European System for Cardiac Operative Risk Evaluation; LVOT, left ventricular outflow tract; MVR, Mitral valve replacement; NYHA, New York Heart Association; POD, postoperative day; STS, Society of Thoracic Surgeons; TAVI, transcatheter aortic valve implantation
